# Hits and misses: leveraging tDCS to advance cognitive research

**DOI:** 10.3389/fpsyg.2014.00800

**Published:** 2014-07-25

**Authors:** Marian E. Berryhill, Dwight J. Peterson, Kevin T. Jones, Jaclyn A. Stephens

**Affiliations:** Program in Cognitive and Brain Sciences, Department of Psychology, University of NevadaReno, NV, USA

**Keywords:** working memory, Gestalt grouping, response inhibition, tDCS, cognitive neuroscience

## Abstract

The popularity of non-invasive brain stimulation techniques in basic, commercial, and applied settings grew tremendously over the last decade. Here, we focus on one popular neurostimulation method: transcranial direct current stimulation (tDCS). Many assumptions regarding the outcomes of tDCS are based on the results of stimulating motor cortex. For instance, the primary motor cortex is predictably suppressed by cathodal tDCS or made more excitable by anodal tDCS. However, wide-ranging studies testing cognition provide more complex and sometimes paradoxical results that challenge this heuristic. Here, we first summarize successful efforts in applying tDCS to cognitive questions, with a focus on working memory (WM). These recent findings indicate that tDCS can result in cognitive task improvement or impairment regardless of stimulation site or direction of current flow. We then report WM and response inhibition studies that failed to replicate and/or extend previously reported effects. From these opposing outcomes, we present a series of factors to consider that are intended to facilitate future use of tDCS when applied to cognitive questions. In short, common pitfalls include testing too few participants, using insufficiently challenging tasks, using heterogeneous participant populations, and including poorly motivated participants. Furthermore, the poorly understood underlying mechanism for long-lasting tDCS effects make it likely that other important factors predict responses. In conclusion, we argue that although tDCS can be used experimentally to understand brain function its greatest potential may be in applied or translational research.

## Introduction

Studies applying transcranial direct current stimulation (tDCS) are growing in frequency due to appealing safety profiles, reasonable cost, and promising findings both for investigating cognition and as a therapeutic intervention. Indeed, a coarse PubMed search combining the search terms of tDCS and publication year 2000 produced four articles, whereas the search in 2013 produced 370 references. TDCS is in wide use in clinical populations such as stroke (Fregni et al., 2005; Hummel et al., 2005; Boggio et al., 2007b; Jo et al., 2009; Kang et al., 2009; Baker et al., 2010; Lindenberg et al., 2010; Chrysikou and Hamilton, 2011; Hamilton et al., 2011), Parkinson's (Boggio et al., 2006; Fregni et al., 2006c), Alzheimer's (Boggio et al., 2011, 2012), depression (Fregni et al., 2006b; Ferrucci et al., 2009; Loo et al., 2010; Kalu et al., 2012), and chronic pain (Fregni et al., 2006a; Lefaucheur et al., 2008). It is also applied to healthy participants in cognitive domains such as working memory (WM) (Marshall et al., 2005; Ohn et al., 2008; Berryhill et al., 2010; Andrews et al., 2011; Mulquiney et al., 2011; Berryhill and Jones, 2012; Jeon and Han, 2012; Jones and Berryhill, 2012; Hoy et al., 2013), episodic memory (Ross et al., 2010, 2011; Javadi and Walsh, 2012; Javadi and Cheng, 2013), perception (Antal et al., 2001, 2003, 2004, 2006; Antal and Paulus, 2008; Bachmann et al., 2010; Bolognini et al., 2011; Borckardt et al., 2012), and motor processing (Nitsche et al., 2005, 2007; Boros et al., 2008; Hunter et al., 2009; Antal et al., 2011).

However, emerging techniques require some measure of trial and error to determine when, where, and how they are best applied. In particular, tDCS faces a number of unknowns with regard to mechanism and implementation that can make experimental design challenging. For instance, short- and long-term mechanisms of tDCS remains poorly understood. Furthermore, there is no standard stimulation protocol intensity or duration (see Nitsche et al., 2008). Thus, the pattern associated with cognitive studies using tDCS is haphazard and difficult to patch together to create a comprehensive snapshot of the literature in a particular domain. In addition, the file-drawer problem may be a particular issue with regard to tDCS. Considerable knowledge may be gained from a more complete airing of these data. The purpose of the present paper is two-fold. In Part 1, we focus on our primary research area, WM, and summarize what WM-tDCS approaches have been successful. Next, we broaden these findings slightly to cognition more generally, although patterns are less clear and the diversity of paradigms and protocols more broad. These collective findings bring several factors to light when considering the use of tDCS to study cognitive questions. In Part 2, we present several studies, both including WM components, one with a primary focus in response inhibition, in which we failed to consider one or more of these factors and failed to observe significant effects. The goal of this article is to facilitate tDCS research in cognition in healthy individuals by sharing what has worked reliably, what has failed, and what lessons we have extracted.

## Part 1: tDCS successes

### What works in working memory?

WM plays a significant role in many cognitive tasks and is controlled by broad frontoparietal networks accessible to tDCS. These features have made it attractive to researchers interested in applying tDCS to studies of cognition. This approach has been successful. The most consistent set of WM data comes from the use of n-back tasks paired with left dorsolateral prefrontal cortex (DLPFC) stimulation in healthy (Ohn et al., 2008; Andrews et al., 2011; Mulquiney et al., 2011; Zaehle et al., 2011) and special populations (Boggio et al., 2006; Jo et al., 2009); for a recent meta-analysis see (Brunoni and Vanderhasselt, 2014); see Table 1. In short, these findings reveal a robust pattern: anodal tDCS to the left DLPFC improves verbal n-back task performance when compared to either sham or cathodal tDCS. This pattern of tDCS-linked WM improvement remains constant across a variety of stimulus intensities, durations, and participant populations. Indeed, recent meta-analysis looking at these studies identified a reliable reaction time improvement during active tDCS in those studies using the n-back and stimulating the DLPFC (Brunoni and Vanderhasselt, 2014, but see also Jacobson et al., 2012).

**Table 1:** **Peer-reviewed studies of WM paired with tDCS**.

**Authors**	**Task**	***N***	**Site**	**mA**	**Dur (min)**	**Comparison**
**IMPROVED WM AFTER ANODAL tDCS TO THE DLPFC: n-BACK TASK**						
Andrews et al., 2011	2-, 3-bk, verbal, digit span	10	L DLPFC, during or before task	1	10	During: digit span: A > S
Berryhill and Jones, 2012	Visual, verbal 2-bk	12^*^, OA	L, R DLPFC	1.5	10	L and R: A > S, in more educated
Boggio et al., 2006	Verbal 3-bk	18, PD	L DLPFC, M1	1, 2	20	DLPFC: 2 mA: A > S
Fregni et al., 2005	Verbal 3-bk	15	L DLPFC, M1	1	10	L DLPFC: A > S
Jo et al., 2009	Verbal 2-bk	10, R. stroke	L DLPFC	2	30	Pre/post differences: A > S
Hoy et al., 2013	Verbal 2-, 3-bk	18	L DLPFC	1, 2	20	RT: A > S: 2-bk: 1 mA faster than 2 mA
Kim et al., 2014	Verbal 3-bk	9/8^*^	L DLPFC	1	20	A > S: *N* = 9 benefited, *N* = 8 no effect
Mulquiney et al., 2011	2-bk, Sternberg	10	L DLPFC	1	10	2-back: A > S
Ohn et al., 2008	3-bk, Korean letters	15	L DLPFC	1	30	Pre/post: A > S
Zaehle et al., 2011	Verbal 2-bk	16	L DLPFC	1	15	A > C
**IMPROVED WM AFTER ANODAL tDCS: MIXED SITES, TASKS**						
Boggio et al., 2009	Visual recognition	10, AD	L DLPFC, L temporal	2	30	L DLPFC: A > S
Jones and Berryhill, 2012	E1. WM Change detection, sequential; E2. WM Change detection	E1: 10^*^; E2: 14^*^	R PPC	1.5	10	E1: High WMC: A,C > S Low WMC; S > A,C; E2: High WMC: A,C > S
Tanoue et al., 2013	Pre-cue, retro-cue WM	23	R PPC, R PFC	1.5	10	Pre-cue: S > C, PPC = PFC; Retro-cue: S > C: PPC > PFC
Tseng et al., 2012	Change detection	10^*^	R PPC	1.5	15	Low WMC: A > S
**IMPAIRED WM AFTER tDCS: MIXED SITES, TASKS**						
Berryhill et al., 2010	Sequential WM, recognition, recall	11	R PPC	1.5	10	Recognition: S > C
Ferrucci et al., 2008	Sternberg	13	Cerebellum	2	15	S > A, C: Impaired practice benefits
Marshall et al., 2005	Sternberg	12	L, R DLPFC	260 μ A	P15	RT: Stim slower than sham

Abbreviations: A, anodal; AD, Alzheimer's disease; bk, back; C, cathodal; Dur, duration of stimulation in minutes; E, experiment; L, left; mA, tDCS strength in milliamperes; N, number of participants; P, pulsed at 15 s on/15 s off, R, right; S, sham; OA, older adults; PD, Parkinson's disease. All findings pertain to accuracy unless reference to reaction time is noted. In cases where the between-subjects effects were significant, N refers to group size and is noted with an ^*^.

This consistency highlights at least one notable exception. When we tested healthy older adults in a verbal and visual 2-back task we replicated the anodal tDCS benefit, but only in those with more education (Berryhill and Jones, 2012). Surprisingly, those with less than a college-degree received no benefit from tDCS. Instead their performance revealed a nearly equal and opposite decrement in performance. Furthermore, regardless of whether the stimuli were verbal or visual and whether stimulation site were the left or the right the differences in education predicted whether tDCS helped or hurt WM performance. This was the first paper, to our knowledge, showing that an inhomogeneous population significantly modulated tDCS effects in different directions. A recent paper has used current modeling to clarify that participants only benefited on a verbal 3-back WM task when the tDCS applying DLPFC during a verbal 3-back task current modeling indicates that those who benefited showed significant modulation in the DLPFC current, but those who did not improved did not show significant DLPFC modulation (Kim et al., 2014). They attributed these data to morphological differences in brain structure and where current flow went. This finding reveals the need for refining stimulation targeting by registering an individual's MRI scans with tDCS electrode placement as is the practice in TMS research. However, their data also showed that those who started with higher WM performance garnered greater benefits from tDCS.

There are several other papers pairing tDCS with different WM tasks (e.g., old/new recognition, recall, change detection) and parietal stimulation sites. These data are less clear. For example, we found that anodal tDCS to the right posterior parietal cortex (PPC: P4) selectively interfered with WM probed by old/new recognition, but not with WM probed by recall (Berryhill et al., 2010). We subsequently found that when participants performed two WM tasks of different difficulty levels in the same session, that tDCS effects were only apparent for the more challenging task (Jones and Berryhill, 2012). Importantly, here, again, there were significant and opposing patterns in the data such that young adults with high WM span benefited but those with low WM span performance was impaired after cathodal or anodal tDCS to the right PPC. In contrast, a similar study also applying anodal tDCS to the right PPC reported that those with low WM performance on the sham day performed better during the anodal session on a *challenging* change detection task (Tseng et al., 2012). However, this study lacked an independent measure of WM capacity to segment their participants. Instead, behavioral performance during the sham session was used. Thus, the observed effects are contaminated by regression to the mean because poor performers during the sham session were likely to perform better at another session regardless of tDCS presence. However, here, again are several data points indicating that population differences predict the direction and magnitude of tDCS effects on WM.

A second issue buried in these data is that effects are apparent when tasks are difficult. Apart from the WM papers just noted there is at least one other analysis that has found that tDCS effects were apparent only when the task demands were difficult. More specifically, in an associative memory task participants learned face-name and place-name pairs and received left or right anodal tDCS. In younger and older adults, tDCS provided a performance benefit only when the participants struggled to produce the correct face or place name, as evidenced by long reaction times (Ross et al., 2010, 2011). One way to think about this in terms of tDCS is that the extra stimulation can serve as a tipping factor. This is consistent with our understanding that tDCS induces changes the changes in neuronal excitability—cells become more (anodal) or less (cathodal) likely to fire action potentials. When tasks are easy, the outcome is clear and the addition of tDCS does not change performance. However, when tasks or even trials are very difficult, tDCS effects emerge. When designing a task to pair with tDCS, it is worth ensuring that the task demands are sufficiently challenging for participants and/or that the more challenging trials can be isolated for separate analysis.

A third emerging issue that becomes more apparent when reviewing the tDCS-WM papers is that the effect sizes tend to be small and the studies are underpowered. For example, although early studies report significant effects with 10 participants, more recent papers tend to include 20 or more participants. One speculation is that early studies tapped homogeneous populations, presumably available lab personnel, to participate and this meant resulted in more consistent performance and tDCS patterns.

A related concern is that as laboratories become more comfortable with the tDCS technique they are subject to added noise from poorly motivated participants. Although this is a problem for many experimental techniques, it may be particularly relevant if unmotivated participants do not engage during challenging tasks or during challenging trials. As mentioned above, the subtle response-shifts induced by tDCS may be particularly sensitive to contamination from poorly motivated participants. However, this notion must be considered as speculative because there are no explicit data testing the role that motivation plays in tDCS designs, although we are currently testing this hypothesis.

### What works in cognition?

Apart from WM, there is broad use of tDCS to investigate wide-ranging cognitive topics (for a recent review see Coffman et al., 2012). A real challenge is the diversity in experimental paradigms and tasks makes it difficult to identify consistent patterns in the tDCS literature. Furthermore, a recent review paper highlighted “foundational” problems associated with tDCS and the impact of variability across participants, issues associated with cognitive set and performance, the reliability of effects over time and current dynamics (Horvath et al., 2014; see also Lopez-Alonso et al., 2014). However, in a few areas of upper-level cognitive domains some consistency is beginning to emerge. In Table 2, we provide an admittedly incomplete survey of cognitive studies employing tDCS reporting significant effects in healthy adults. As in WM, the majority of cognitive studies target frontal stimulation sites and thus, it may not be surprising that the papers describing significant effects of tDCS relate to upper-level cognitive tasks (e.g., response inhibition, memory, decision making). From these scattered findings, it can be difficult to predict the direction of effects in a tDCS study and it can be difficult to know why the effects are as they are. As noted above, these are the studies with positive findings and likely there are many other null findings that would be informative for the research population. In the following section, we raise several points to consider when developing tDCS studies of cognition.

**Table 2:** **Peer-reviewed studies of cognitive questions in healthy adults paired with tDCS**.

**Authors**	**Task**	**N**	**Site**	**mA**	**Dur (min)**	**Comparison**
**ENHANCED PERFORMANCE AFTER tDCS**						
Cerruti and Schlaug, 2009	Semantic memory (verbal associates)	E1: 18; E2: 12	L, R DLPFC	1	20	L DLPFC A > C, S
Chi et al., 2010	Visual episodic memory	12^*^	Bilateral R/L anterior T	2	13	L C/R A:>S
Fecteau et al., 2007	Risk-taking	E1: 10^*^; E2: 6^*^	Bilateral oppositional DLPFC; E2 L DLPFC	2	15	Bilateral < S, unilateral Lower risk taking
Fecteau et al., 2012	Deception	12^*^	Bilateral oppositional DLPFC	2	20	RT: Active < S
Floel et al., 2008	Language learning	19	L A, C, sham peri-sylvian	1	20	A > S
Iyer et al., 2005	Speed, emotion, verbal fluency	E2: 43 (1 mA), 30 (2 mA)	L DLPFC	1, 2	20	2 mA: A > S verbal fluency
Jacobson et al., 2011	Response inhibition	11	R and oppositional bilateral inferior frontal gyrus;	1	10	RT: A < S
Karim et al., 2010	Guilty Knowledge Test	E1: 22; E2: 22	Anterior PFC	1	13	C > S, deceptive behaviors
Kincses et al., 2004	Probabalistic learning	22	L PFC, V1	1	10	A PFC > S, implicit learning
Mameli et al., 2010	Guilty Knowledge Task, Visual Attention	20	Bilateral A DLPFC	2	15	RT: guilty knowledge: A < S
Marshall et al., 2004	Word-pair memory	E1: 18	Bilateral A, sham PFC during slow-wave sleep	0.26	30	A > S
Ross et al., 2010	Face/Place-name memory	15	L, R anterior T	1.5	15	R A > S: face/name pairs
De Vries et al., 2010	Artificial grammar	19^*^	Broca's area	1	20	A > S
Penolazzi et al., 2014	Retrieval induced forgetting (RIF)	20^*^	R DLPFC	1.5	20	C removed RIF
Mungee et al., 2014	Conditioned fear memory	37^*^	R PFC	1	20	A > S; *stronger* fear
**IMPAIRED PERFORMANCE AFTER tDCS**						
Boggio et al., 2010	Gambling	9–10^*^	Bilateral L/R DLPFC	2	15	L A/R: C > S riskier
Stone and Tesche, 2009	Local/global attentional switching	14	L PPC	2	20	S > A, C
Tanoue et al., 2013	Pre-, retro- attentional cueing	23	R PFC, PPC	1.5	10	S > C; Pre: PPC = PFC; Retro: PPC > PFC
**MIXED EFFECTS AFTER tDCS**						
Boggio et al., 2008	Response inhibition	14	L A T, R C T	2.0	13	Active vs. S: Women < errors, Men: > errors
Boggio et al., 2009	Rating valenced images	23	L M1, DLPFC, occipital	2.0	5	L DLPFC: A < S pain ratings
Bolognini et al., 2010	Visual search	10^*^	E1: R PPC; E2: L PPC	2.0	20	R PPC: A > S training gains
Knoch et al., 2008	Ultimatum bargaining	30 C, 34 S	R PFC	1.5	10	C > S: Reduced punishment of unfair behavior

Abbreviations: A, anodal; C, cathodal; Dur, duration of stimulation in minutes; E, experiment; L, left; M1, primary motor cortex; mA, tDCS strength in milliamperes; N, number of participants; R, right; S, sham; T, temporal lobe; V1, primary visual cortex. All findings pertain to accuracy unless reference to reaction time (RT) is noted. In cases where the between-subjects effects were significant, N refers to group size and is noted with an ^*^.

## Considerations when applying tDCS to cognitive questions

Here, we summarize some of our observations as a set of factors to consider before using tDCS in a cognitive experiment. We also provide some rationales for strategically violating these recommendations while remaining successful.

### Homogeneous populations

Equal and opposite effects in different populations can easily obscure effects. Researchers interested in relating structure-function relationships can reduce the noise in their data by targeting a homogeneous population. More practically, armed with the knowledge that population differences are pertinent it is advisable to include measures of relevant factors such as WM capacity in a WM study. This permits incorporating some demographic or other factor in the analysis. This is particularly relevant for applied and translational applications of tDCS in development for general use. For this purpose, it is essential to identify population differences and use that information to predict who will garner the greatest benefit from tDCS.

### Low power

The effect sizes in cognitive studies of tDCS are modest. Thus, it is important to counter low power by enrolling sufficiently large cohorts. Because population differences have been reported showing equal and opposite effects of tDCS, it is likely others exist. Such differences may be adding significant noise and obscuring positive findings.

### Challenging tasks

The effects of tDCS are subtle. It is unlikely that tDCS could significantly influence supraliminal response patterns. As such, it is during near-threshold events that tDCS effect become apparent—for instance, when the task is really difficult. Experimental design should include tasks that are adaptive such that all participants are performing an effortful task. Analyses should be designed to permit separation of easy trials (e.g., high accuracy, fast responses) from more difficult trials.

### Poor motivation

This issue is related to the use of challenging tasks. We suspect that tDCS is particularly sensitive to participants with low motivation. This may be a particular problem when testing freely available undergraduate volunteers who value course credit more than the research. Low motivation may matter because tDCS effects are subtle and seem to shape performance only over the range in the heart of response functions where there is variability in the response outcome. In other words, tDCS will not change someone's response when it is 100%, but it may push responses from 49% in one direction to 52%.

## Part 2: proving the point

Below we give examples for which we have concluded our criteria were not adequately met. We offer them in the hopes that our missteps will permit others to avoid them.

### Grouping mechanisms in visual WM

#### Fault: poor motivation

One area of interest for tDCS relates to improving function, in particular visual WM (VWM). Some reports suggest that VWM can benefit from Gestalt principles of grouping (e.g., proximity, similarity, connectedness, common fate) as they facilitate visual perception (Wertheimer, 1950; Palmer and Rock, 1994). Specifically, incorporating similarity, proximity, common fate, common region, or uniform connectedness improves VWM performance in change detection tasks (Xu, 2002, 2006; Woodman et al., 2003; Xu and Chun, 2007, 2009; Brady and Tenenbaum, 2013; Peterson and Berryhill, 2013; Luria and Vogel, 2014). Moreover, a recent fMRI experiment found evidence that grouped arrays were associated with lower amplitude responses in the BOLD signal corresponding to the intraparietal sulcus (IPS) during maintenance when compared to ungrouped items (Xu and Chun, 2007). The inferior parietal regions that reflect increases in set size up to VWM capacity limits (e.g., Todd and Marois, 2004, 2005; Xu and Chun, 2006) register grouped items as intermediate steps rather than as full set size increases.

Consequently, we hypothesized that tDCS targeting the right IPS would modulate grouping benefits associated with VWM performance. Our previous work had already identified VWM disruption after cathodal tDCS (1.5 mA, 10 min) to this same parietal site (Berryhill et al., 2010; Tanoue et al., 2013). Specifically, we predicted that cathodal tDCS would interrupt VWM grouping. We also anticipated that the interruption would be more pronounced in those with high WM capacity as preliminary data showed that these individuals benefited from grouping more than low WM capacity individuals. Furthermore, we had previously identified enhanced tDCS effects for challenging tasks in high WM capacity individuals (Jones and Berryhill, 2012). Thus, we anticipated the possibility of observing different patterns of effects as a function of high or low WM capacity.

### Method

#### Participants and tDCS protocol

Thirty-three right-handed, neurologically intact graduate and undergraduate students with normal or corrected-to-normal vision participated in the current experiment (Mean age = 21.3, 25 female) and received $15 per hour. On separate days, participants completed the VWM task after sham or cathodal tDCS (right posterior parietal scalp site: P4, 10 min, 1.5 mA; Eldith Magstim GMbH, Ilmenau, Germany). During the sham session, current was ramped up and down for the first and last 20 s of the stimulation interval to mimic sensations associated with current change. The anode was placed on the contralateral cheek. Session order was counterbalanced across participants. Active/sham stimulation occurred prior to the task, while participants completed practice trials. Electrodes were removed prior to the start of the task. All experimental protocols were approved by the Institutional Review Board of the University of Nevada.

#### Stimuli

The task design slightly modified a paradigm previously published that showed significant VWM grouping benefits (Xu and Chun, 2007). We summarize these methods here. Stimuli were gray, symmetrical novel shapes (2.6 × 2.6° visual angle) presented on black rectangles (6.9 × 18° of visual angle) against a gray background. There were three experimental conditions varying the stimulus grouping: 2-ungrouped (2 shapes in separate black rectangles), 3-ungrouped (3 shapes in separate black rectangles), and 3-grouped (2 shapes in one black rectangle and one in a separate rectangle). The stimuli were presented at 57 cm using EPrime® (Psychology Software Tools, Sharpsburg, PA) software running on a Dell © desktop computer and were presented on a 20.5″ by 13″ widescreen monitor running at a refresh rate of 60 Hz.

#### Procedure

**Figure 1:**
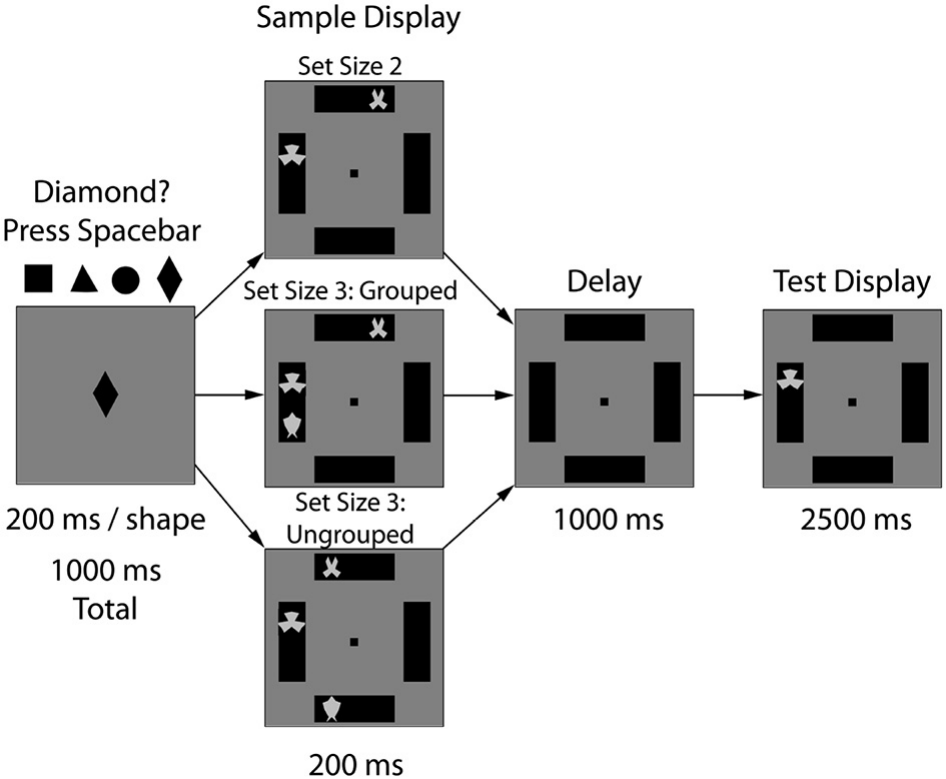
**Experiment 1 task design**. After 10 min of sham or 1.0 mA anodal tDCS to the right PPC (P4), the experimental task began. Participants viewed three types of arrays: 2 items (2-ungrouped), 3 items in two boxes (3-grouped), or 3 items in three boxes (3-ungrouped). After a WM delay period (1000 ms), a single probe item appeared and participants indicated whether that stimulus had previously appeared in that location.

To insure focused attention, trials began with a fixation task (1000 ms), in which participants viewed a rapidly changing shape (e.g., triangle, circle, square, diamond, 200 ms/item) and were required to make a key press response each time the diamond shape appeared; see Figure 1. Next, the stimulus display of the VWM task appeared (200 ms), followed by a delay (1000 ms), and finally a single probe item (2500 ms). Participants were instructed to press the “o” key if the probe item had been shown in the same location during the stimulus array (50%) and the “n” key if the probed item changed from sample to test. A feedback display followed (1300 ms: “Correct” in blue font, “Incorrect” or “No Response Detected” in red font). Participants completed 15 practice trials to familiarize themselves with the task prior to beginning the experiment and completed 150 experimental trials (50 trials per condition). During the sham session, the forward and backward digit spans were administered as an independent measure of WM capacity (e.g., combined digit span score; WAIS-IV, Wechsler, 2008). The experimental task lasted approximately 20 min.

### Results

**Figure 2:**
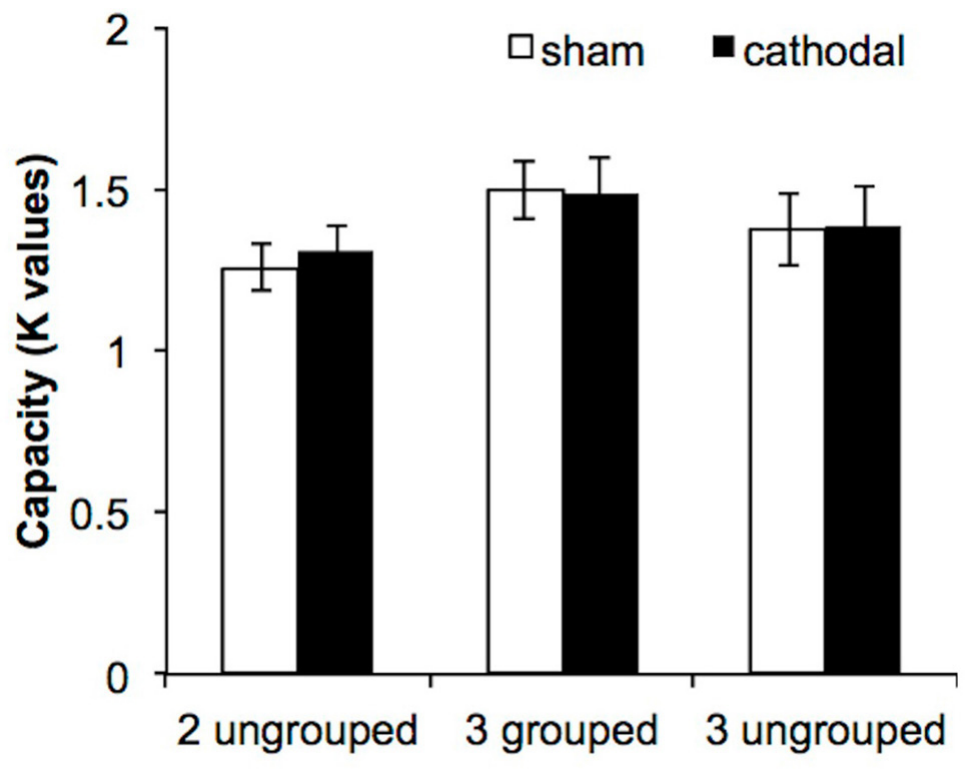
**VWM performance in Experiment 1**. The three experimental conditions are displayed along the abscissa, while estimated capacity (based on Cowan's *K* formula, 2001) is depicted along the ordinate. The error bars represent the standard errors of the means in each condition for each tDCS session.

For each condition and each participant we estimated WM capacity by calculating Cowan's *K*: [*K* = set size^*^ (hit rate—false alarm rate)]. The first question was to determine whether cathodal tDCS to the right parietal lobe interfered with WM grouping mechanisms. The *K*-values were subjected to repeated-measures ANOVA with the factors of condition [2-ungrouped (2-UG), 3-grouped (3-G), 3-ungrouped (3-UG)] and tDCS session (active, sham). The data replicated the expected behavioral grouping benefit [*F*_(2, 64)_ = 5.39, *p* = 0.007, η^2^_*p*_ = 0.14, β = 0.83] such that capacity was significantly larger in the 3-G condition compared to the 2-UG condition (3-G = 1.49, 2-*UG* = 1.29, *p* = 0.004, Bonferroni corrected here and throughout); see Figure 2. Additional pairwise comparisons indicated that there were no differences between the 2-ungrouped and 3-ungrouped conditions (3-UG = 1.38, *p* = 0.26) or between the 3-ungrouped and 3-grouped conditions (*p* = 0.44). In other words, grouping numerically enhanced performance on the 3-grouped condition, but not significantly. However, the answer to the primary question was that there was no significant effect of tDCS on VWM performance as there was no main effect of tDCS [*F*_(1, 32)_ = 0.07, *p* = 0.79] and no significant condition × tDCS interaction [*F*_(2, 64)_ = 0.18, *p* = 0.83].

Our concerns regarding participant homogeneity prompted dividing participants into high and low WM capacity groups based on the independent WM measure of combined forward + backward digit span scores (high *N* = 16: combined digit span score >13; low *N* = 17: combined digit span score <13). The inclusion of WM capacity in the ANOVA did not change the pattern of the results. The grouping benefit remained [*F*_(2, 62)_ = 5.26, *p* = 0.008, η^2^_*p*_ = 0.15, β = 0.82]. As before, pairwise comparisons reveled significant differences between the 2-ungrouped and the 3-grouped condition (*p* = 0.005), but not between the 2-ungrouped and the 3-ungrouped conditions (*p* = 0.26) or between the 3-grouped and the 3-ungrouped conditions (*p* = 0.47). As before, there was no main effect of tDCS [*F*_(1, 31)_ = 0.05, *p* = 0.83], and no two- or three-way interactions (all *p*'s > 0.13). In summary, there was no difference in VWM performance in response to grouping via common region between high and low WM capacity individuals. Additionally, diverging from our predictions, there was no effect of cathodal tDCS relative to sham tDCS.

### Discussion

Here, we tested the hypothesis that cathodal tDCS targeting parietal regions involved in grouping processes would disrupt these processes. Contrary to our predictions, tDCS did not modulate grouping benefits in VWM. Furthermore, WM capacity did not contribute to the current results as cathodal tDCS failed to interrupt grouping benefits to WM performance in either high or low WM capacity participants. These findings were unexpected because we previously interrupted WM using cathodal tDCS to the right parietal lobe in a VWM recognition task (Berryhill et al., 2010). One possibility to account for these null results is that participants may not have been effortfully engaged in the task. This interpretation is supported by low VWM performance (e.g., mean K-values for the 2-UG, 3-UG, 3-G conditions: 1.29, 1.38, 1.49 items compared to the ~1.9, 2.3, 2.6 items per condition reported by Xu and Chun, 2007). Anecdotally, in our previous successful study, the participants were largely graduate students known to the experimenter, rather than undergraduates interested in obtaining course extra-credit.

Alternatively, previous fMRI findings revealed bilateral IPS activity during this task (Xu and Chun, 2007). Thus, some might argue that a unilateral stimulation protocol might not have sufficient power to prevent some contralateral compensatory mechanism. However, we think that this is unlikely for several reasons. First, there is evidence supporting right IPL in attending to stimuli across both visual hemifields (e.g., Sheremata et al., 2010; Szczepanski and Kastner, 2013), making it more likely to see disrupted performance in the VWM task after right lateralized stimulation. Second, and perhaps of greater relevance, we previously interrupted VWM using the identical tDCS protocol but different VWM tasks (Berryhill et al., 2010; Tanoue et al., 2013). Thus, we suspect that participants' engagement was the most important factor in this particular experiment.

### Reducing ADHD impulsivity

#### Faults: low power, heterogeneous population, low task difficulty

The familiar symptoms of attention deficit hyperactivity disorder (ADHD) include impulsivity, restlessness, and difficulty concentrating (Faraone and Biederman, 2005). Recent findings suggest that there is abnormal brain structure and function in the pre-supplementary motor area (pre-SMA) in ADHD. When people with ADHD perform tasks requiring response inhibition they have smaller activations in the pre-SMA (Mulligan et al., 2011). These findings suggest that the pre-SMA is not sufficiently activated during response inhibition tasks. Recently, in healthy adults response inhibition was predictably modulated by tDCS to the pre-SMA: anodal improved performance whereas cathodal tDCS impaired performance (Hsu et al., 2011). Other reports show tDCS-linked improvement in response inhibition tasks in those with major depressive disorder (Boggio et al., 2007a) and stroke (Kang et al., 2009). Thus, we tested whether directing anodal tDCS to the pre-SMA would modulate performance in a response inhibition task, the Go/No-Go task. First, based on the logic just described, we anticipated that anodal tDCS would improve Go/No-Go task performance in young adults with low or high ADHD symptomology, and thereby replicating the Hsu et al. findings in healthy young adults using the stop-signal task (Hsu et al., 2011). We anticipated an interaction such that those with high symptomology would garner greater tDCS benefits than the low symptomology group. We also completed two WM tasks: the operation span task and a spatial n-back task. These tasks were included to clarify the specificity of tDCS influences. Both WM tasks engage frontoparietal networks, but were not expected to show modulation by tDCS to the pre-SMA. Here, we tested an unmedicated undergraduate population to look at ADHD symptomology because we do not apply tDCS to people taking stimulants or anti-depressants (e.g., those prescribed for ADHD).

### Method

The University Institutional Review Board approved all protocols. Volunteers completed the Adult ADHD Self-Report Scale (ASRS—v1.1; Kessler et al., 2005). This short screen was developed to identify ADHD symptomology in adults and has been validated in adult (Adler et al., 2006) and college-aged (Fuller-Killgore et al., 2013) populations. Scores were derived from the six-screener questions in Part A of the ASRS, the most predictive of ADHD. Questions probed the frequency with which participants forgot appointments, completed tasks, or felt distracted Responses were converted from verbal labels (e.g., “never,” “sometimes,” “rarely,” “often,” “very often”) to point values (1–5). Questions 1–3 required doubling the point value when answers of “sometimes,” “often,” or “very often” were recorded; questions 4–6 required doubling the point value when answers “often” or “very often” were recorded. Four or more answers requiring doubling meet the criteria for “high likelihood” of ADHD (Kessler et al., 2005). To identify participants we recruited participants high (ASRS scores 39–58) and low (ASRS scores <19) in ADHD symptomology. This high ADHD group met the criteria of high likelihood of ADHD. Thirty-six right-handed, normal, neurotypical participants were subsequently enrolled (age 18–37, 14 male). Participants were screened to ensure they were not taking medications that modulate the excitability of the brain (e.g., stimulants). Participants completed anodal, cathodal and sham tDCS sessions, in counterbalanced order across 3 separate days with a minimum washout period of 24 h. Current was administered using a commercial stimulator (Eldith Magstim GMbH, Ilmenau, Germany). To target the right pre-SMA, we placed the electrode 2 cm to the right of FZ (10–20 system) and the reference electrode was placed on the contralateral cheek. Participants received 10 min of 1.5 mA tDCS during anodal and cathodal sessions and during sham stimulation current was ramped up and down for the initial and final 20 s of the period. Participants completed practice trials of each task during stimulation and after the electrodes were removed, they began the experimental trials of the tasks described below.

#### Behavioral tasks

***Go/No-Go Task***. In this measure of response inhibition and impulsivity participants view a letter stream (400 ms) and respond when they see a target letter (“x” or “y”) *unless* the same target preceded the current target letter. In other words, in the sequence “x-t-c-b-x-g-y,” the first “x” and the “y” require responses, but the second “x” requires response inhibition. One to six distractor letters were presented between each target letter. There were 440 target letter trials and the task lasted approximately 12 min.

***Automated Operation Span (OSpan)***. The OSpan requires participants to solve arithmetic problems while also maintaining letters in WM (Unsworth et al., 2005). Participants report remembered letters after completing arithmetic problems. The task consisted of 9 sets of letters and arithmetic problems and lasted approximately 10 min.

***Spatial 2-back***. Participants completed a spatial 2-back WM task in which a green circle (4.8°, 500 ms, ITI: 1500 ms) was presented in 9 possible locations. Participants reported whether the current item matched what was shown two-items previously via button press (match: “j,” non-match: “f”). Participants completed 450 trials (150 match; 300 non-match), which lasted approximately 15 min.

### Results

**Figure 3:**
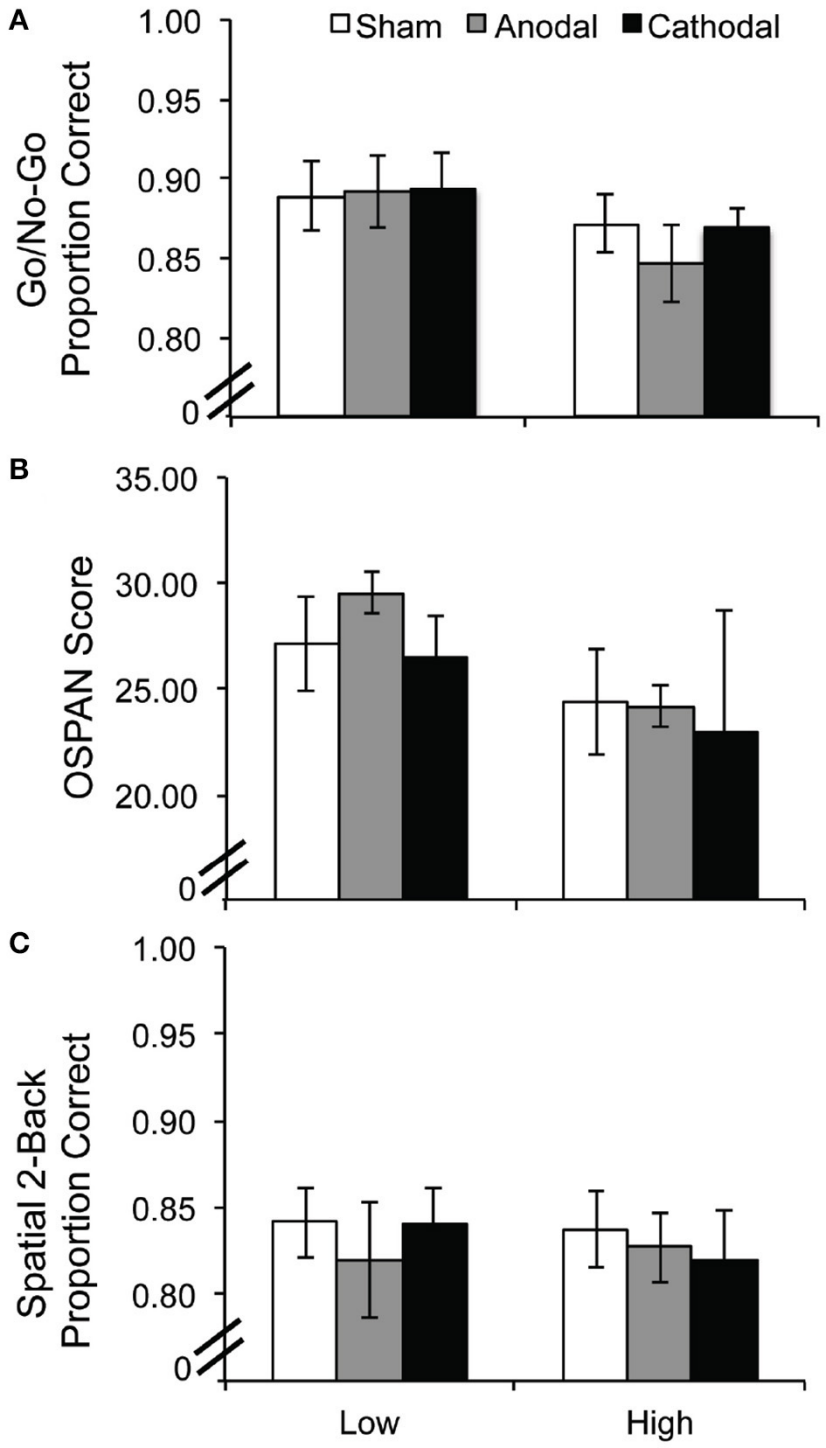
**Experiment 2 performance per task as a function of tDCS condition and ADHD symptomology: (A) Go/No-Go, (B) OSPAN, and (C) Spatial 2-back task**. Error bars represent the standard error of the mean.

Performance on each task was subjected to a 2 ADHD group (low, high symptomology) × 3 tDCS condition (sham, anodal, cathodal) repeated measures ANOVA. Performance accuracy on the Go/No-Go task revealed no significant main effect of group [*F*_(1, 17)_ = 1.01, *p* = 0.33], no main effect of tDCS [*F*_(2, 34)_ = 0.996, *p* = 0.38], and no interaction [*F*_(2, 34)_ = 1.145, *p* = 0.33]; see Figure 3A. The reaction time data were also analyzed and they followed the same pattern of null results {group [*F*_(1, 17)_ = 0.246, *p* = 0.626]; tDCS [*F*_(2, 34)_ = 0.060, *p* = 0.942], interaction [*F*_(2, 34)_ = 1.643, *p* = 0.208]}. Four participants were eliminated from the OSPAN and 2-back WM tasks because they pressed the same button for all responses. Performance on the OSPAN task followed the same pattern, with no significant main effects of group [*F*_(1, 15)_ = 1.635, *p* = 0.22], or tDCS [*F*_(2, 30)_ = 0.151, *p* = 0.860], and no interaction [*F*_(2, 30)_ = 0.213, *p* = 0.810]; see Figure 3B. This pattern was also true for performance accuracy on the spatial 2-back WM task. There were no main effects of group [*F*_(1, 15)_ = 0.015, *p* = 0.904] or tDCS [*F*_(2, 30)_ = 0.466, *p* = 0.632] and no significant interaction [*F*_(2, 30)_ = 0.750, *p* = 0.481]; see Figure 3C.

### Discussion

Here, we failed to replicate an anodal tDCS benefit on a response inhibition task performance after stimulation to the pre-SMA (Hsu et al., 2011), and to extend it into those with high/low ADHD symptomology using the go/no-go task. Others have had luck with response inhibition and tDCS, particularly in suppressing responses (reviewed in Juan and Muggleton, 2012). We may have tapped a particularly heterogeneous population and used an insufficiently difficult response inhibition task that did not allow us to discriminate between easy and challenging trials. We may have selected a stimulation site that was too lateralized and an intensity that did not effectively reach the pre-SMA. These issues may have clouded tDCS effects. Future studies should include a more difficult response inhibition task, as participants approached ceiling. In the Hsu et al. (2011) paper, the stop-signal task they employed required more finely titrating the timing of the stop signal to ensure that the task difficulty was tailored to the participant. Finally, because the participants completed a self-assessment rather than complete a clinical interview and ADHD diagnosis, a different pattern of results may emerge in a clinically diagnosed ADHD population.

In the two WM tasks, the OSPAN and spatial 2-back tasks, likewise, we did not observe any significant modulation after tDCS. These later results were expected, but difficult to interpret as site-specific tDCS effects given the null findings in the primary task of interest, the go/no-go task.

## General discussion

Neurostimulation via tDCS is a useful tool for investigating aspects of cognition. As with any tool there are some things that work better than others, and a set of appropriate situations for a given tool. Here, we have summarized some findings demonstrating successful applications of tDCS in studies of WM (see Table 1) and other cognitive modalities (see Table 2). Although these studies all report positive findings there is still considerable variability in terms of the pattern of effects, paradigms used and tDCS parameters. For instance, stimulus intensity, duration, tDCS electrode montage are inconsistent. The most consistent pattern in the published literature has been to report significant improvements in WM tested in verbal n-back tasks and anodal tDCS to the left DLPFC. In other cognitive realms a patchwork of findings is emerging revealing consistent effects in memory, deception, and cognitive control. However, there are exceptions and forays into different tasks, populations, and parameters have produced different patterns of results. We also note that the proliferation of neurostimulation effects (e.g., tACS, TRNS) will be certain to raise additional issues to maximize their experimental and applied usefulness (for a recent review see Kuo and Nitsche, 2012; see also Snowball et al., 2013).

We think the file-drawer problem is a particular challenge here. We offered several of our own missteps and several factors to consider when applying tDCS to cognitive questions. We believe that appropriate consideration of these factors will facilitate future experimental design and serve to increase interpretable outcomes. Ideally, the following is available: a homogenous, highly motivated, large population engaging in a challenging cognitive task that permits selective identification and analysis of the most difficult trials. When one or more of these factors is overlooked there may be little to report.

### What do these considerations reveal about tDCS?

The considerations we noted above point us toward an emerging research question. Why do group differences matter? The underlying mechanism of tDCS remains unclear, although long-term neuroplasticity is indicated (Rosenkranz et al., 2000; Nitsche et al., 2003). Some pharmacological work in humans indicates that blocking sodium or calcium channels prevents longer-lasting effects of anodal tDCS and antagonizing NMDA receptors prevents longer-lasting effects of anodal or cathodal tDCS, at least in motor cortex (Nitsche et al., 2003). Furthermore, at a larger scale, the way current flows through a particular individual's brain certainly varies and has tremendous implications for applied and experimental use of tDCS (Bikson et al., 2013). Incorporating other variables, including the structural and functional connectivity of stimulated networks and their temporal dynamics will also improve experimental success when applying tDCS. We suspect that the answers to these questions will also clarify why some groups benefit more than others and why homogeneous groups provide more consistent data. For an individualized approach to tDCS application, the underlying molecular mechanism must be fully clarified and this in turn will advance our understanding at the network level. At this point, another way to counter these differences is simply through brute power and by running sufficient participants.

The role of task difficulty is important. As noted in the introduction, when a participant knows the answer definitively tDCS is unlikely to have an effect. This means tasks that are too easy do not show any effect of tDCS. It is only in the challenging, borderline cases where tDCS serves as a tipping factor. As we age, we encounter more of these near-threshold cases and this population may be the one to benefit most from tDCS-linked cognitive interventions. In particular, in the realm of WM, small improvements can benefit quality of life and are worth the effort to gain them.

## Conclusions

tDCS is a safe, affordable neurostimulation technique that is well-tolerated in healthy and special populations. Regular tDCS is not well-positioned to target carefully defined cortical regions for studies of structure-function relationships. The current article provided several considerations for review when preparing a tDCS study. Because tDCS provides the greatest effect at threshold level decisions, challenging tasks are necessary. Participants must be sufficiently engaged and motivated. Finally, the nature of the participants also should be carefully considered as group differences can predict nearly equal and opposite responses to tDCS. Furthermore, whereas early tDCS studies often targeted motor and visual regions, studies of cognition require sufficient power and larger numbers of participants. When these considerations were sufficiently weighed, we have succeeded. We offer several cautionary tales detailing our failed efforts when these considerations were ignored. Finally, in conclusion, the best use of tDCS may well be in translational and applied settings. In this arena, tDCS already shows promise as a way to maintain and/or restore cognitive function across various populations. Clearly, there is great interest in effective cognitive interventions as the aging population grows and tDCS may be a part of the answer.

### Conflict of interest statement

The authors declare that the research was conducted in the absence of any commercial or financial relationships that could be construed as a potential conflict of interest.
